# *In utero* position matters for littermate cell transfer in mice: an additional and confounding source with maternal microchimerism

**DOI:** 10.3389/fimmu.2023.1200920

**Published:** 2023-07-28

**Authors:** Mathilde Giassi, Marie F. Hemon, Marielle Martin, Jean Roudier, Isabelle Auger, Nathalie C. Lambert

**Affiliations:** ^1^ Institut National de la Santé et de la Recherche Médicale (INSERM) Unité Mixte de Recherche (UMRs) 1097 Arthrites Autoimmunes, Aix Marseille Université, Marseille, France; ^2^ Arthritis R&D, Neuilly-sur-Seine, France; ^3^ Rheumatology Department, Assistance Publique des Hôpitaux de Marseille (AP-HM), Marseille, France

**Keywords:** chimerism, *in utero*, littermate, maternal, HLA-DR4, mouse

## Abstract

**Introduction:**

Feto-maternal cell transfer during pregnancy is called microchimerism (Mc). Its persistence in respective hosts is increasingly studied as to its potential role in immune tolerance, autoimmunity, cancer, and degenerative diseases. Murine models with transgenic reporter genes, heterozygously carried by the mother, allow maternal Mc tracking in wild-type (WT) offspring. However, as gestation in mice is multi-embryonic, an exchange of cells between fetuses carrying the same reporter gene as their mother and negative WT littermate, named littermate Mc (LMc), can occur and be confounded with the maternal source. We propose here to evaluate LMc contribution in mice.

**Methods:**

To avoid the maternal confounding source of Mc, transgenic males, heterozygous for a reporter gene, here, the human leukocyte antigen DRB1*04 (DR4^+/−^), were crossed with WT females (DR4^−/−^). DR4^+/−^ LMc was specifically quantified by HLA-DR4 quantitative PCR, i) *in utero* in main organs from 15 DR4^−/−^ fetuses from three litters of 11, nine, and five; and ii) after birth in two litters of eight pups: in two DR4^−/−^ stillborns and four DR4^−/−^ adult mice.

**Results:**

At embryonic stages, DR4^−/−^ fetuses having one or two nearby DR4^+/−^ littermates in the same uterine horn were almost seven times more frequently positive for DR4− microchimerism in their organs (*p* = 0.01) and had quantitatively more LMc (*p* = 0.009) than those without nearby DR4^+/−^ littermates. Furthermore, LMc persists at birth and into adulthood with interindividual heterogeneity.

**Conclusions:**

This study identifies heterogeneity for LMc acquisition according to *in utero* position and different interpretation of previously published results on maternal Mc in mice.

## Introduction

We must forget the dogma that the placenta is a hermetic barrier. Maternal cells migrate into the fetus via the placenta and vice versa (1). Donor cells (maternal or fetal) persist in peripheral blood and various organs of their respective host (2, 3). These phenomena are called maternal microchimerism (MMc) and fetal Mc.

MMc was initially detected in infants with severe combined immunodeficiency (SCID) (4), but it soon became apparent that it could be observed, although at a lower level, in immunocompetent infants and adults (2, 5).

MMc has been reported in the human fetal circulation as early as 13 weeks of gestation (6) and in the murine fetal circulation at 13 days of gestation to reach its maximum between the 17th and 19th day (7).

In humans, it has been identified in fetal lymph nodes, where it plays a role in inducing early tolerance to maternal antigens in the offspring (8). Such tolerogenic response toward non-inherited maternal antigens (NIMA) has long-term consequences. For example, organ or bone marrow transplants donated by haploidentical siblings to recipients, mismatched for the NIMA, have a better outcome than when mismatched for the non-inherited paternal antigen (NIPA) (9, 10). In other words, the recipient has been “educated” *in utero* and has kept immunological tools to tolerate maternal cells/antigens for decades.

MMc has also raised interest in the context of human autoimmune diseases. Maternal cells have been found in the pancreas of children with type 1 diabetes or hearts of infants with neonatal lupus after cardiac block without a clear establishment of whether these cells repair or destroy the colonized tissue [for the complete review, refer to (11)].

However, the limited access to human fetal and adult organs somewhat limited MMc research and led to the use of animal models. Having the same type of hemochorial placenta as humans and gestations of only 21 days, mice are a “good model” to study Mc.

For the last decades, various mouse models have been designed to generate detectable genetic differences between mother and fetus [for review, refer to (12)]. Very often murine models with transgenic reporter genes (tg), heterozygously carried by the mother (tg^−/+^), are used to track MMc in wild-type offspring (tg^−/−^). However, gestation in mice is generally multi-embryonic. As a result, an exchange of cells between the different embryos, defined here as littermate Mc (LMc), can occur and be confounded with the maternal source. Nevertheless, some embryos carrying the same reporter gene as their mother can transfer reporter-positive microchimeric cells (tg^−/+^) to a negative wild-type littermate.

In this study, to avoid the maternal confounding source of Mc, we carried out opposite mouse crosses, such that only the LMc could be detected in the progeny to evaluate its real impact. We crossed a transgenic male mouse heterozygous for a reporter gene, here the human leukocyte antigen DRB1*04 (DR4^+/−^), with a wild-type female (DR4−/−). This combination allowed us to specifically quantify LMc (DR4^+/−^) in the main organs of DR4^−/−^ fetuses constituted at embryonic stages between E15.5 and E18.5 and in 13 different organs of 5-month-old adult mice.

## Materials and methods

### Mouse strains and crosses

DBA/2JRccHsd (DBA/2) mice were purchased from Envigo Laboratories (Indianapolis, IN, USA). Major histocompatibility complex (MHC) haplotype antigens of DBA/2 mice were H2^d/d^. C57BL/6NRj (C57BL/6) mice were purchased from Janvier Labs (Le Genest-Saint-Isle, France). MHC haplotype antigens of C57BL/6 mice were H2^b/b^. Knock-out/knock-in (KO/KI) C57BL/6NRj mice were made by replacing I-E^b^ genomic DNA (KO) with gene fragments containing HLA-DRA and HLA-DRB1*04*04 (KI) (H2^b/b^ DR4^+/+^) as previously described (13) (CIPHE, Marseille, France).

By crossing DBA/2 females (H2^d/d^) and C57BL/6 KOKI*04:04 (H2^b/b^ DR4^+/+^) males, we could obtain heterozygous B6-DR4D2F1 (H2^d/b^ DR4^+/−^) males used for crossbreeding experiments as detailed below.

Six DBA/2 (H2^d/d^) females (named DBx #1 to #6) were mated with an heterozygous H2^d/b^ DR4^+/−^ male. From these crosses, three DBA/2 females (DBx #2, 4, and 5) were sacrificed during gestation to test LMc in the fetuses, and three DBA/2 females (DBx #1, 3, and 6) were left to give full-term birth to test LMc in adults, but pups from DBx #1 were cannibalized at birth.

We could then obtain fetuses or adults negative for DR4 (DR4^−/−^) and fetuses or adults DR4^+/−^ heterozygous that had inherited the paternal DR4 haplotype.

Thus, in gestational DBA/2 females, the only DR4^+^ source of Mc received by the DBA/2 (H2^d/d^ DR4^−/−^) embryos comes from nearby DR4^+/−^ littermates.

The day of vaginal plugging was considered day 0 of gestation. The three females were planned to be sacrificed on the 18th day of gestation to have fetuses whose organs are sufficiently distinct and formed. One female (DBx #4) was miscalculated as to her gestational day and was thus sacrificed earlier than planned [gestational day 15.5 according to the anatomic appearance of the embryo (14)].

All mice used for crosses were 7 to 9 weeks old and weighed 20–30 g. Mice were housed at the Luminy INSERM Institute, Marseille (A1301303). All experiments were approved by the animal ethics committee (*APAFIS #3266-201512181131807v4* and *APAFIS #22865-2020112517402887 v1*). All animal care and experimental procedures were performed in agreement with the Animal Ethics Committee of Marseille and the Ministère de l’Enseignement Supérieur et de la Recherche.

### Organ sampling in fetuses

**Figure 1:**
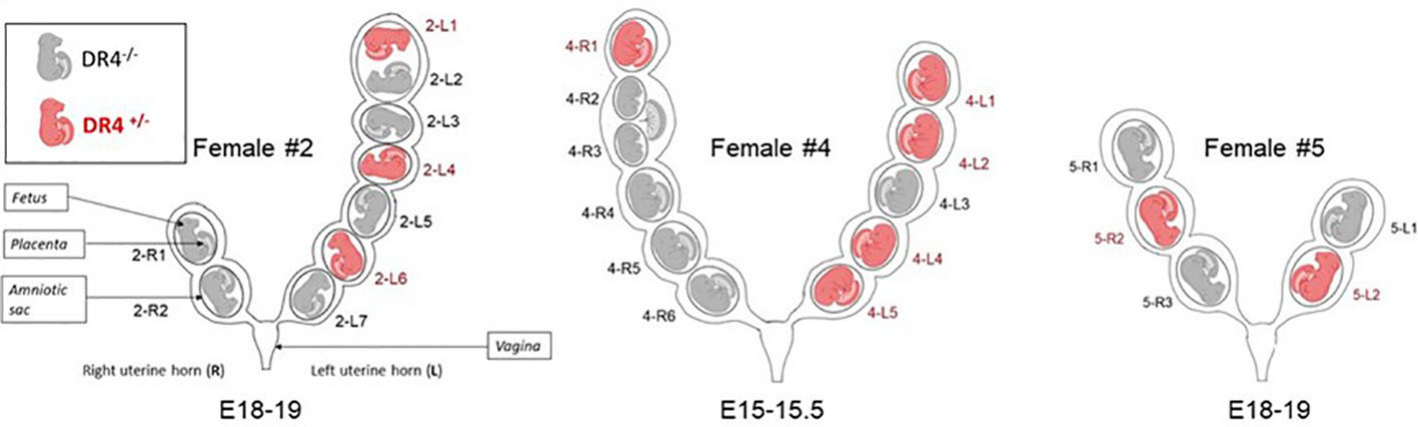
Schematic representation of uterine horns from the three gestant DBA/2 females having been crossed with a C57BL/6-KOKI DR4^+/−^ male. Fetuses are named according to their right (R) or left (L) position in the uterine horn and numbered from the top of the uterine horn (rostral position) to the bottom (caudal position). For example, 2-R1 fetus is a fetus from female #2 in the right uterine horn in the upper position. Fetuses were genotyped by HLA-DR4 PCR to distinguish those that had inherited the paternal DR4. DR4^+/−^ fetuses are shown in red and DR4^−/−^ fetuses in gray. Of note, female #2 carried two embryos sharing the same amniotic sac (2-L1 and 2-L2) but with individual placentae. Female #4 carried embryos sharing the same placenta (4-R2 and 4-R3) but with individual amniotic sacs.

Three DBA/2 gestant female mice (Dbx #2, #4, and #5) were anesthetized with isoflurane, then euthanized in a CO_2_ chamber, and dissected to collect the uterine horns. The fetuses were collected and named according to their right (R) or left (L) position in the uterine horn (**Figure 1**) and numbered from the top of the uterine horn (rostral position) to the bottom (caudal position). Each fetus was carefully dissociated, keeping the amniotic sac intact to avoid any contamination of other fetuses. Once the fetus was placed on a clean dissecting board, the amniotic sac was removed. The placenta was collected, and the embryo was dissected to isolate different organs (thymus, heart, lungs, pancreas, liver, kidneys, skin, and brain). Scissors and forceps were carefully cleaned between each isolated organ within the same individual to avoid cross-contamination. Fetuses from the gestational age E15.5 did not have the organs as well constituted as the E18-19 fetuses; thus, mostly only the liver and the brain were systematically collected, and the remaining fetal tissue was directly extracted and called “remaining tissue” (**Table 1**).

**Table 1:** Quantification of littermate Mc in different organs from fetuses according to their distant (Group 1) or proximal (Group 2) position to a DR4+ source of Mc.

	Fetus ID	Gestation timing	Number of DR4+ fetuses nearby	Number of DR4 littermate microchimeric cells (LMc) per million of host cells expressed in genome equivalent of cells per million (gEq/10^6^)											
				Placenta		Thymus	Heart	Lung	Pancreas	Liver	Right kidney	Left kidney	Skin	Brain	Remaining tissue
Group 1	2-R1	E18-19	0	0		0	8	0	0	0	0	0	0	0	
	2-R2	E18-19	0	0		0	0	0	0	0	0	0	0	0	
	4-R3	E15-15.5	0	0						0				0	0
	4-R4	E15-15.5	0	5						0				0	0
	4-R5	E15-15.5	0	0			0			0				0	0
	4-R6	E15-15.5	0	0						0				0	0
Group 2	2-L2	E18-19	1	46		5	0	5	0	0	0	0	11	5	
	2-L3	E18-19	1	201		0	0	0	6	0	6	5	51	0	
	2-L7	E18-19	1	6		0	0	0	5	0	0	0	4	0	
	4-R2	E15-15.5	1	0						0				0	3
	5-R1	E18-19	1	6		0	0	0	0	0	0	0	0	0	
	5-R3	E18-19	1	19		0	0	0	0	0	0	0	0	4	
	5-L1	E18-19	1	0		5	0	0	0	0	0	0	0	0	
	2-L5	E18-19	2	46		0	0	0	0	0	0	0	381	0	
	4-L3	E15-15.5	2	2703			5			67				36	14

p-Values are calculated between groups.

na, not applicable because of the small number of individuals in one of the groups. ns, not significant.Cells are formatted in color scale, with lower positive LMc values in light orange and higher values in dark orange.

### Organ sampling in adults

**Figure 2:**
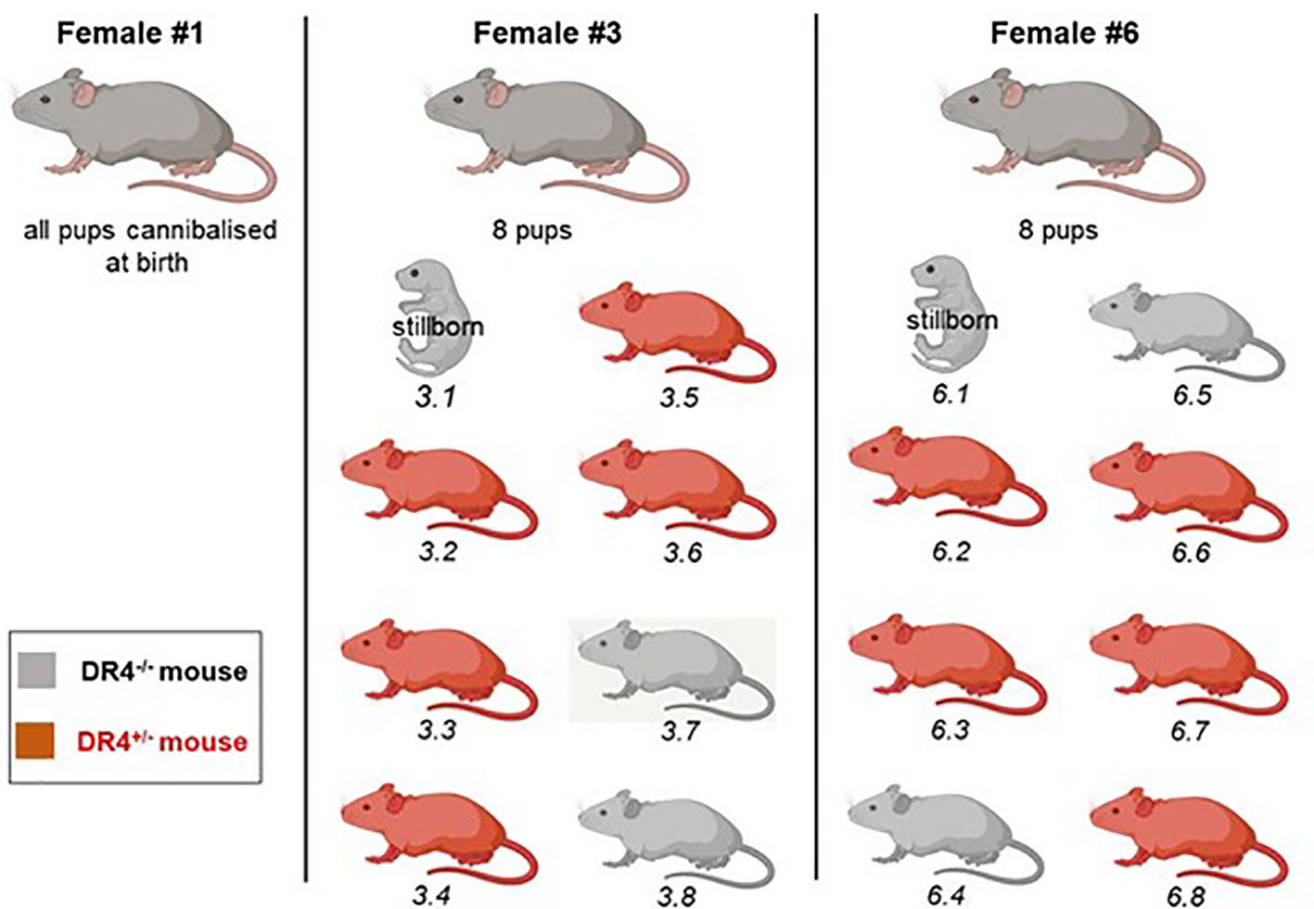
Representation of litters from three DBA/2 females crossed with a C57BL/6-KOKI DR4^+/−^ male. Female Dbx #1 ate her pups at birth. Females Dbx #3 and #6 each had a stillborn pup and seven others. Genotyping was realized from a blood draw after weaning to indicate whether the pup had inherited or not the paternal DR4 haplotype. Mice carrying the DR4 paternal haplotype are shown in red, and mice negative for the DR4 paternal haplotype in gray.

Three DBA/2 gestant female mice (Dbx #1, #3, and #6) were isolated in a separate cage prior to giving full-term birth to not confound pups from the different litters and to test LMc in offspring to adulthood. All pups from Dbx #1 were cannibalized. Dbx #3 and #6 had eight pups each including one stillborn in each litter (**Figure 2**). The two stillborns were genotyped and tested for LMc, as they were both DR4^−/−^. The remaining 14 pups were left to grow and were annotated and genotyped after weaning. At 5 months old, they were anesthetized with isoflurane, then euthanized in a CO_2_ chamber, and dissected to collect organs/tissues with similar cautious cleaning of instruments described above. In addition to the blood drawn from the mandibular vein prior to euthanasia, 13 organs/tissues were collected: thymus, heart, lung, pancreas, liver, right kidney, left kidney, skin, brain, ovaries, bone marrow, lymph nodes, spleen, and muscles.

### Purification and quantification of genomic DNA from tissues

Genomic DNA was extracted from tissues with the EZ1&2 DNA Tissue Kit (Qiagen, Valencia, CA, USA) using the EZ1^®^ Advanced XL instrument (Qiagen) according to the manufacturer’s instructions.

The total concentration of genomic DNA in each sample was evaluated by amplification of the β actin gene, using a standard reference curve, prepared from commercialized mouse DNA (Promega, Madison, WI, USA) ranging from a genome equivalent DNA (gEq) of 70,000 cells (70,000 gEq) to a gEq of 1 cell (1 gEq), with the conversion factor being ~6.6 pg of DNA per cell. Forward 5′-ACTCATCgTACTCCTgCTTgCT-3′ and reverse 5′-AgCTCACCATTCACCATCTTgT-3′ primers and 5′FAM-ATggAgCCACCgATCCACACAgA-3′TMR probe were synthesized by TIB MolBiol (Berlin, Germany) and used at 300 and 100 nM, respectively. Quantitative TaqMan^®^ PCR was performed using Light Cycler FastStart DNA MasterPLUS reaction kits on a LightCycler^®^480 instrument (Roche Diagnostics, Basel, Switzerland). The amplification conditions consisted of an initial incubation at 95°C for 10 min and 45 cycles of 95°C for 15 s and 60°C for 1 min. Data were analyzed using LightCycler software version 3.5.3.

### Identification of DR4^+/−^ or DR4^−/−^ genotype of the descendants

The β actin standard curve also served as a reference to prepare the standard curve for HLA-DRB1*04 (*HLA-DR4)* quantification assay. To confirm DR4^+/−^ or DR4^−/−^ genotypes, extracted DNA from embryonic or adult tissues was screened by *HLA-DR4* quantitative PCR assay according to the previously described method and following the same amplification conditions except for an annealing temperature of 62°C (15).

Fetuses or adult mice, tested in duplicates for which the equivalent DNA of cells evaluated by β actin corresponded to the same equivalent of cells tested by HLA-DR4 PCR, were the ones that had received the paternal DR4 haplotype and were DR4^+/−^. Therefore, they could not be tested for littermate DR4 Mc.

Fetuses or adult mice negative or very slightly positive for DR4 PCR (due to possible Mc) were the ones that had not received the paternal DR4 haplotype and could be tested for littermate DR4 Mc on further wells of DNA for all tissues (see **Supplementary Figure S1**).

### Quantification of DR4^+/−^ LMc

In regard to the β actin standard curve, all DNA samples were adjusted to an optimal concentration close to 20,000 gEq with a maximum at 30,000 gEq (**Supplementary Table S1**), as the optimal sensitivity for Mc detection was 1 gEq of microchimeric cells in 20,000 gEq of host cells (0.005%). Samples were tested for Mc in 10 or more replicate wells for a combined final concentration of approximately 200,000 gEq and a combined final maximal sensitivity of ~0.0005%.

Samples for which chimerism was found were systematically verified for their amplification curve (see all results in **Supplementary Figure S2**). As we used TaqMan probes and not DNA-binding dyes such as Sybr-Green, artifact PCR amplifications due to mispriming or self-hybridization of the primers, resulting in erroneous amplification products or primer–dimers, were circumvented. Thus, a sample was considered positive even when only one well out of 10 had an amplification, as soon as the amplification curve was typical of an amplification. Negative controls were systematically found negative, thus excluding sample contaminations. When results calculated from the software were given a number of Mc cells inferior to 1, the result was arbitrarily rounded up to 1.

### Statistical analysis

Statistical analyses were conducted using GraphPad Prism 9.1.2 software (La Jolla, CA, USA). Chi-two (χ^2^) or Fisher’s exact test was used to compare the frequency of fetuses being positive for LMc between two groups: the group of fetuses with no nearby DR4^+/−^ fetuses (Group 1) and the group of fetuses with one or two nearby DR4^+/−^ fetuses in the same uterine horn (Group 2). The non-parametric Mann–Whitney test was used to compare quantities of LMc between the two groups. A *p*-value <0.05 was considered significant.

## Results

### Fetus genotyping

To determine whether cells can transfer between mouse littermates, we have crossed heterozygous B6-KOKIDR4-D2 F1 males (H2^d/b^ DR4^+/−^) with DBA/2 females (H2^d/d^) (named DBx #2, #4, and #5). Such crosses allowed that the only source of DR4+ Mc received by the DBA/2 (H2^d/d^ DR4^−/−^) fetuses from pregnant DBA/2 females comes from nearby DR4^+/−^ littermates and not from maternal Mc (**Supplementary Figure S3**). We then genotyped by HLA-DR4 specific Q-PCR all the fetuses to determine which one had inherited the paternal DR4 gene (unfit for DR4 Mc research) and which one did not (suitable for DR4 Mc research). The HLA-DR4 primers and probes are specific and do not cross-react with any other HLA-DR genes or murine MHC.

Of the 25 fetuses studied from the three different pregnant DBA/2 females, 10 carried the paternal DR4 gene, and the remaining 15 were negative for DR4 genotyping and could be tested for DR4^+/−^ LMc (**Figure 1**, fetuses in gray color). DBA/2 females #2, #4, and #5 had respectively six, six, and three fetuses testable for LMc.

### Differences in quantities of LMc cells among individual fetuses

HLA-DR4 Q-PCR was used to quantify LMc on the 116 organs dissected from the 15 DBA/2 fetuses. For each organ tested, Mc results were issued from 10 wells of approximately 15–20,000 gEq cells tested. The mean equivalent number of cells tested for LMc for each organ is detailed in **Supplementary Table S1**. Results are expressed as the number of gEq of Mc cells per million of host cells.

To test whether the proximity to a DR4^+/−^ littermate allows larger quantities of Mc to be transferred to its DR4^−/−^ neighbor, compared to a distant situation, we have divided the fetuses into two groups: the group of fetuses with no nearby DR4^+/−^ fetus (Group 1) and the group of fetuses with one or two nearby DR4^+/−^ fetuses on the same uterine horn (Group 2) (**Table 1**). Thus, fetuses 2R1, 2R2, 4R3, 4R4, 4R5, and 4R6 were in Group 1, and fetuses 2L2, 2L3, 2L5, 2L7, 4R2, 4L3, 5R1, 5R3, and 5L1 were in Group 2 (see **Figure 1**). Of note, in Group 2, fetuses 2L5 and 4L3 were surrounded on both sides by DR4-positive fetuses and had the highest levels of LMc observed in the placenta (4-L3), liver (4-L3), skin (2-L5), brain, or remaining fetal tissue (4-L3).

Fetuses having one or two nearby DR4^+/−^ littermates in the same uterine horn had more frequently DR4 LMc in their placenta (two-tailed Fisher’s test, *p* = 0.04) than those without nearby DR4^+/−^ littermates and had quantitatively more LMc (Mann–Whitney test, *p* = 0.001) (**Table 2**). However, what is seen in the placenta may not transmigrate into the fetal tissues. Then, we separately analyzed organs specific to fetuses (thymus, liver, etc.). Fetuses from Group 2 had more frequent DR4 LMc in any of their organs than those from Group 1 (*χ* ^2 = ^6.83, *p* = 0.01) and had quantitatively more LMc (Mann–Whitney test, *p* = 0.009) (**Table 2**).

**Table 2:** Statistical differences in frequency and quantity of littermate Mc between the two groups of fetuses.

Littermate microchimerism comparison between fetuses from Group 1 and Group 2											
Differences per organ											
	**Placenta**	**Thymus**	**Heart**	**Lung**	**Pancreas**	**Liver**	**Right kidney**	**Left kidney**	**Skin**	**Brain**	**Remaining Tissue**
Frequency	*P=0.04*	*na*	*na*	*na*	*na*	*ns*	*na*	*na*	*na*	*ns*	*na*
Quantity	*P=0.01*	*na*	*na*	*na*	*na*	*ns*	*na*	*na*	*na*	*ns*	*na*
Differences all organs confounded excluding placenta											
Frequency		*P=0.01*									
Quantity		*P=0.009*									

*Stillborn mouse in which whole blood could not be drawn and all organs could not be collected.nd: not done.

### LMc is more often found in the skin

The placenta is the richest tissue with LMc. Indeed, of the nine H2^d/d^ fetuses close to at least one H2^d/d^ DR4^+/−^ embryo, seven (78%) have a microchimeric placenta with a median amount of 46 gEq/10^6^. Although the placenta is expelled at birth and is not part of the fetuses’ organs, strictly speaking, fetuses with high levels of LMc in the placenta are the ones that had received more LMc in their organs. The organ the most often perfused with LMc was the skin with 57% of fetuses close to an H2^d/d^ DR4^+/−^ embryo and with a median quantity of 31 gEq/10^6^. However, the difference was not statistically significant in the skin compared to the other organs. Finally, 3/9 fetuses (33%) had LMc in the brain, confirming a previously described crossing of the blood–brain barrier.

### No massive transfer of LMc from one uterine horn to the other

We have arbitrarily defined the groups as having or not a DR4 nearby fetus on the same uterine horn, but one could wonder whether LMc could be transferred from the right to left uterine horn or vice versa. Only fetuses that have no other possibility of acquiring DR4^+^ Mc than from the other uterine horn can be used to test this hypothesis. Fetuses 2-R1, 2-R2, and 4-R6, falling within these criteria, were systematically negative in the 24 organs tested except fetus 2-R1, which had 4 gEq of Mc cells per million of host cells in the heart. These results do not exclude a slight intra-horn passage of cells but exclude a massive transfer.

### Persistence of LMc at birth and in the adult mice

To determine whether LMc could persist in the adult mice, three DBA/2 females (DBx #1, 3, and 6) were left to give full-term birth to test LMc in adults. Pups from DBx #1 were cannibalized at birth. Females Dbx #3 and #6 each had a stillborn pup and seven others (**Figure 2**). In each litter, the stillborn and two adults were H2^d/d^DR4^−/−^ and could be tested for LMc. The two stillborn mice were positive for LMc with noticeably high levels in the skin of #6.1 (**Table 3**). Out of the four adults tested, one had 8 gEq/10^6^ of LMc in the thymus and the same quantity in the pancreas.

**Table 3:** Quantification of littermate Mc in different organs and whole blood from adult or stillborn mice from two different litters.

				Number of DR4 littermate microchimeric cells (LMc) per million of host cells, expressed in genome equivalent of cells per million (gEq/10^6^)														
		Age months	Number of DR4+ individuals in the litter	Blood	Thymus	Heart	Lung	Pancreas	Liver	Right kidney	Left kidney	Skin	Brain	Ovaries	Bone marrow	Lymph nodes	Spleen	Muscles
**Female #3**	3.1*	**5**	**5**	*nd*	0	0	0	0	0	**19**	0	0	0	*nd*	*nd*	*nd*	0	*nd*
	**3.7**	**5**	**5**	0	0	0	0	0	0	0	0	0	0	0	0	0	0	0
	**3.8**	**5**	**5**	0	0	0	0	0	0	0	0	0	0	0	0	0	0	0
**Female #6**	6.1*	**5**	**5**	*nd*	0	**7**	0	**12**	0	0	0	**848**	0	*nd*	*nd*	*nd*	**4**	*nd*
	**6.4**	**5**	**5**	0	0	0	0	0	0	0	0	0	0	0	0	0	0	0
	**6.5**	**5**	**5**	0	**8**	0	0	**8**	0	0	0	0	0	0	0	0	0	0

*Stillborn mouse in which whole blood could not be drawn and all organs could not be collected.nd: not done.Cells are formatted in color scale, with lower positive LMc values in light orange and higher values in dark orange.

## Discussion

Trafficking of cells between mother and fetus during a healthy pregnancy is well described in humans and animals (1, 16, 17). Similarly, although less documented, cell exchange between twins has been confirmed in many case reports in humans and also in cows or littermate pigs (18–20). Long-term persistence of natural microchimerism in the host has important immune consequences even if the number of persisting cells is relatively low when compared to the iatrogenic chimerism obtained after transplantation.

Indeed, a low number of cells are able to make a detectable biological contribution, such as the production of interleukin-2 (IL-2) in IL-2 KO mice (IL-2^−/−^) born to heterozygous mothers (IL-2^+/−^) (21). IL-2-expressing maternal cells passing through the placenta are able to produce the cytokine in amounts detectable by conventional methods (21). However, when studying maternal Mc with the mouse as a model, one must be warned that maternal and littermate Mc can be confounded because of multi-embryonic gestation in this animal. In this particular mating configuration where the dam is heterozygous for the reporter transgene (tg^+/−^) used to track MMc in transgene-negative offspring (tg^−/−^), some littermates may also have inherited the maternal gene and transfer tg^+/−^ microchimeric cells to other fetuses negative for this gene, in which case LMc can be mistaken for MMc. Therefore, several studies in mice reporting the presence of MMc in offspring cannot definitely rule out the presence of LMc [for review, refer to (12)], unless they have, like in the study of Zhou et al. (22), i) used a fetus derived from a GFP^(−/−)^ female that had been irradiated, reconstituted with GFP^(+/−)^ bone marrow cells, and then mated with a GFP^(−/−)^ male, or ii) used fetuses derived from GFP^(−/−)^ fertilized eggs that had been transferred into the uterus of GFP^+^ foster mothers (oocyte transfer), or iii) used a mother homozygous for the reference transgene and tested the presence of MMc (tg^+/+^) by flow cytometry.

To our knowledge, only one study has evaluated LMc in mice, although it was not the main purpose of that study (23), and it was in adult mice. After crossing a heterozygous male H2^b/d^ with a homozygous H2^b/b^ female and looking at the transfer of littermate H2^b/d^ cells into H2^b/b^ siblings, Dutta et al. concluded that the amounts of LMc, present only in heart tissues from three littermates out of 16, were negligible with quantities of 1 gEq per 10^5^ cells (23).

In the current study, using a transgenic male mouse heterozygous for a reporter gene, here, the HLA-DRB1*04 (DR4^+/−^), with a wild-type female (DR4^−/−^), we similarly specifically evaluated and quantified LMc and demonstrated a consequent exchange of cells between littermates *in utero.* We have expressly chosen to first analyze this transfer *in utero* to better see whether the exchange depends on the proximity to the source of Mc. Indeed, we show that fetuses having one or two nearby DR4^+/−^ littermates in the same uterine horn were almost seven times more frequently positive for DR4 LMc in their organs and had statistically higher quantities of LMc than those that did not have a DR4 fetus nearby.

A limitation of our study could be that the fetuses from the group without nearby DR4^+/−^ fetuses (Group 1) were often from mouse #4, which had been involuntarily dissected at an earlier stage of gestation (E15.5) than the two other mice. Consequently, because the organs were less well constituted at this stage, not all of them could be removed and analyzed for LMc, which could have led to a bias in the results. However, when analyzing data from placentae from both groups—placenta being the organ systematically taken and analyzed for each fetus—results are highly convincing and unequivocal with a stringent difference between the two groups.

Being strongly irrigated by the blood flow from the uterine artery, the placenta was logically the most microchimeric organ with levels reaching up to 0.3% of total cells. This high quantity was observed in particular in a placenta from a fetus surrounded on both sides by two DR4^+^ fetuses (#4-L3, **Figure 1**). An interesting observation published in 1992 by Vom Saal et al. using an intracardial injection of carbon dyes showed that the circulatory flow of the uterine artery and the uterine vein in mice is bidirectional with a rostral flow coming to vascularize the fetuses in the highest position and a caudal flow coming to vascularize those in the lowest position (24). Both flows generally meet in the middle of the loop, depending upon the number of fetuses to vascularize (**Supplementary Figure S4**). For example, the left uterine horn of mouse #4, with five embryos, should have a rostral and caudal flow merging on the central embryo (#4-L3 in this example) draining rostral and caudal DR4 Mc, which may explain the overload of Mc in this embryo. This type of observation, with some embryos being overloaded in LMc, could explain the results obtained in the study from Fujimoto et al. where MMc and LMc were not distinguished and where most embryos had a comparable number of “MMc” cells, while in rare cases, a much higher number of “maternal” cells could be detected (25).

The placenta is however an ephemeral organ that is quickly expelled at birth. Thus, the quantities of Mc received in the placenta are only the ultimate reflection of what can be transmitted to the other organs. We can clearly see that the fetuses for which the placenta is rich in LMc have also an enrichment in the other organs, in particular the skin, where LMc preferentially condenses or migrates.

It is particularly interesting that the skin is one of the target organs of LMc, as a few cases of fraternal microchimerism involved in skin conditions, such as lichen planus or childhood dermatosis, have been described in humans (26, 27). For example, male Mc has been detected in blood and lesional skin biopsy samples from a 9-year-old girl who had an HLA-identical dizygotic twin brother. She was affected with a rare ulcerative acral variant of lichen planus, a skin disease of unknown cause with T cell-mediated inflammation of the epidermis (26). Our group has also demonstrated the presence of female cells from a vanished twin as a source of Mc in a 40-year-old scleroderma-like male with a history of professional exposure to hydrocarbons at the age of 20 who developed skin and lung inflammation within months after the toxic exposure (28).

Whether LMc or fraternal Mc preferentially migrates to the skin remains to be proved with more tested animals, but clearly, the current study shows that more than half of the fetuses (57%) with a nearby source of Mc had LMc in the skin. Moreover, among the mice we planned to analyze in adulthood, two were stillborn, and one of them had remarkable amounts of LMc also in the skin. At this stage of the study, we have no idea why such high amounts are observed in this mouse (848 genome equivalent of LMc cells per million of host cells). We also do not know if the fact that both stillborns have LMc, while this is less often observed in adults, is the cause or the consequence of their death. Further studies need to be conducted to see whether inadequate placental function (mediated by growth restriction and hypoxic injury), which generally causes stillbirth, could generate higher traffic or accumulation of LMc in the dead host.

Another interesting point, already pointed out with fetal Mc in mice and humans (29, 30) or maternal Mc in mice (31), is that LMc can cross the blood–brain barrier, as a third of fetuses having a DR4 littermate nearby had DR4^+^ LMc in their brain.

It has been shown in a very recent study, where MMc was accurately identified, that maternal cells are involved in the offspring’s developing brain by promoting microglia homeostasis, early brain wiring, and behavioral development (31). It seems very likely that LMc and MMc would have different biological consequences on their hosts since the former originates from embryonic/immature cells, while the latter originates from adult cells, but this remains also to be demonstrated.

As expected, because of the rather compartmentalized circulation between the right and left uterine horns (24), we did not observe a massive transfer of LMc from one uterine horn to the other (see **Supplementary Figure S4**). Only the equivalent DNA of 4 cells per million host cells was observable in the heart of one of the three fetuses tested for inter-horn passage of cells. Although we cannot completely exclude the false-positive signals from contamination, it seems unlikely it happened as we have been extremely meticulous during the collection procedure by cleaning the dissecting instruments between each fetus and between each isolated organ within the same individual. It seems therefore more likely that these few Mc cells are the result of a very slight inter-horn passage.

Finally, although a limitation of our study was that we could only test four 5-month-old adult mice, we could find LMc in one of them, and interestingly, LMc was present in the thymus, suggesting that this Mc source may have an immune influence in the host.

In humans, of course, multi-embryonic pregnancies are not as usual as in mice. Twinning varies with several maternal characteristics including age, ethnicity, nutrition, and fertility (32, 33), but overall, it occurs in about 1/80 of pregnancies, which is not that rare (34). Furthermore, the phenomenon of vanishing twin, which goes unnoticed most of the time, is relatively common in healthy pregnancies and could greatly increase this proportion (34). Twinning should thus be considered as a possible source of pathogenic microchimeric cells in humans, in male patients with autoimmune diseases where female cells are detected (35), or in nulliparous women where male cells are detected (36).

In conclusion, mouse models remain an essential tool in elucidating the functional role of MMc, but we must keep in mind that LMc can be a confounding source of Mc and that previously published results on MMc in mice may be differently interpreted. Our study highlights interindividual differences for LMc in fetuses according to their position *in utero*, interindividual differences, which can be found later in adults. In other words, microchimeric cells from littermates increase genetic diversity in mice with the same genetic background depending on their *in utero* proximity to the source. This may be of great importance for immune responses and explain interindividual heterogeneity.

## Data availability statement

The original contributions presented in the study are included in the article/**Supplementary Material**. Further inquiries can be directed to the corresponding author.

## Ethics statement

The animal study was reviewed and approved by Animal Ethics Committee of Marseille and the Ministère de l’Enseignement Supérieur et de la Recherche APAFIS# 3266-201512181131807v4 APAFIS #22865-2020112517402887 v1.

## Author contributions

MH, MG, and NL designed the research. MH, MG, MM, IA, and NL performed experiments. MH, MG, IA, JR, and NL analyzed data. NL wrote the paper, and NL and MG provided the drawings and figures. All authors contributed to the article and approved the submitted version.
