# Supplementary material for: In utero position matters for littermate cell transfer in mice: an additional and confounding source with maternal microchimerism
Source: Front Immunol. 2023 Jul 28;14:1200920. doi: 10.3389/fimmu.2023.1200920 (PMC10422045; doi:10.3389/fimmu.2023.1200920)
Supplement: Supplementary file 1 [file DataSheet_1.docx]

**Supplementary Figures and Tables**

**
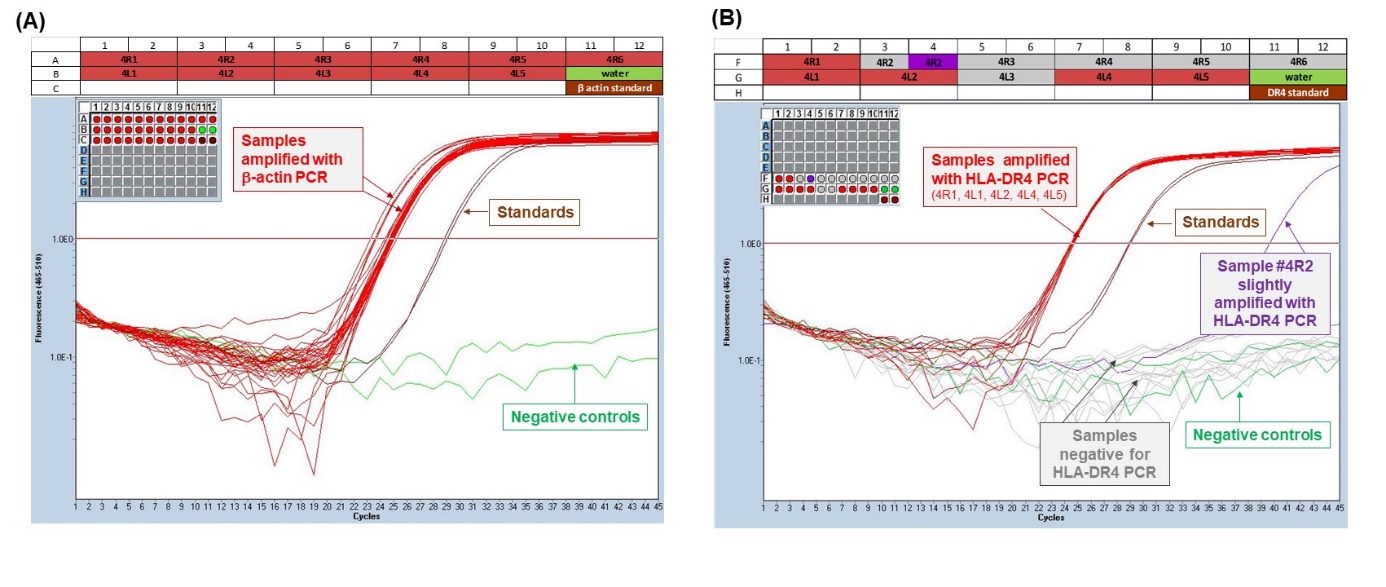
**

**Figure S1. Genotyping of fetuses by HLA-DR4 specific Q-PCR**

**(A)** DNA samples from all fetuses were first tested with β actin PCR (house-keeping gene) to evaluate DNA concentration, all samples were positive with values ranging between 31,200 and 43,200 gEq of cells, and were further adjusted to Mc -specific-QPCR concentrations; **(B)** DNA samples from all fetuses were then tested with HLA-DR4 PCR. Samples positive for DR4 (in red) were the one who had received the paternal DR4 haplotype. Being DR4^+/-^, they could not be tested for littermate DR4 Mc. Fetuses negative (in grey) or very slightly positive (in purple) for DR4 PCR (sample #4R2) were the one who had not received the paternal DR4 haplotype and could be tested for littermate DR4 Mc on further tissue samples.


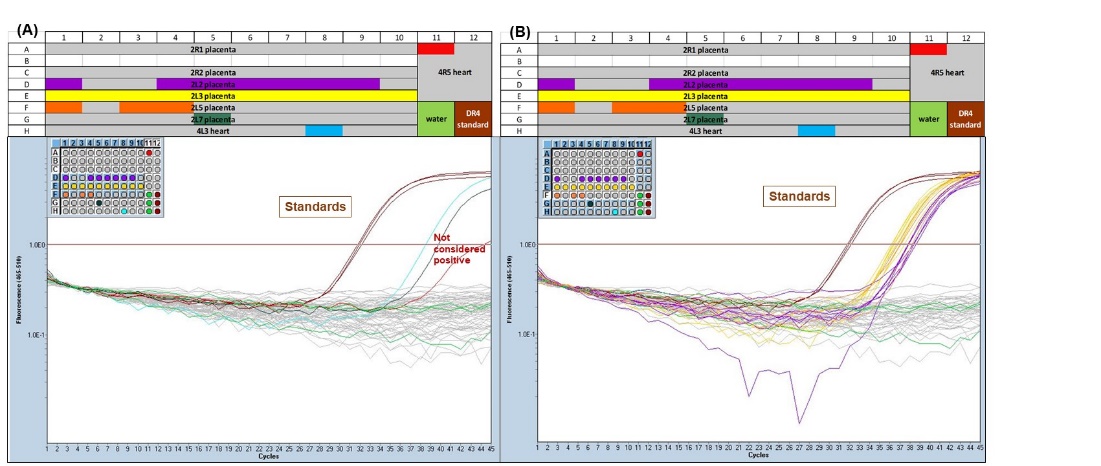


**Figure S2. Amplification curves of microchimeric HLA-DR4 positive samples.**

*Standards are represented in burgundy/brown, negative controls with water in green, amplified samples are in any other color and non-amplified samples in grey.*

1. **
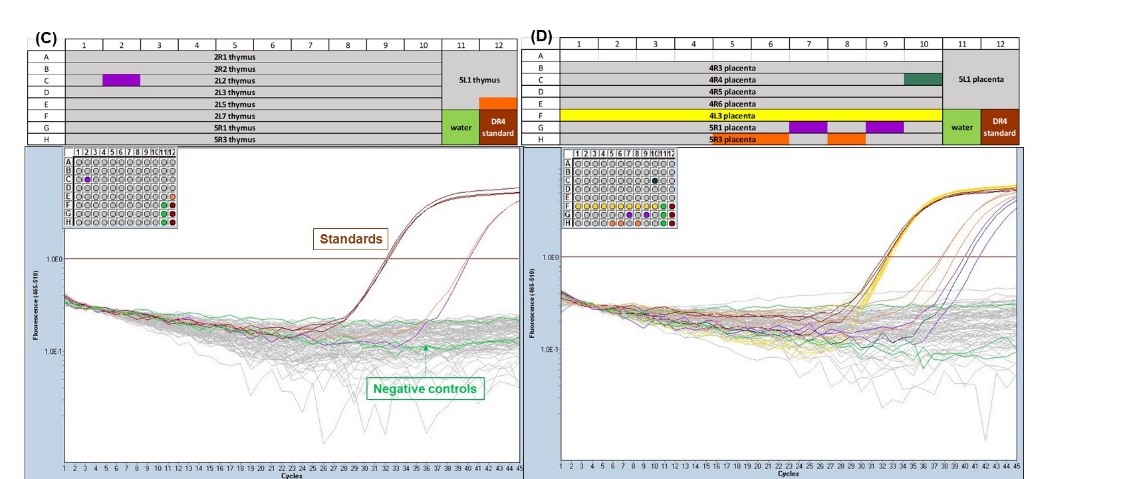
***In this PCR plate three samples had low amplifications in one well out of the ten tested : sample #2L7 in the placenta (dark green well and curve), sample #4L3 in the heart (turquoise blue well and curve) and sample #4R5 in the heart (red well and curve). Nevertheless, the far-right red curve from sample #4R5 was not considered as an amplification curve because of its shape, thus not considered as positive.* ***(B)*** *Samples from the same plate giving high levels of LMc are represented on this separate picture to prevent the amplification curves of the samples with the highest levels of LMc from hiding those with very low. Amplifications curves of all HLA-DR4 positive samples represented in* ***(C)****,* ***(D)****,* ***(E)****,* ***(F)****,* ***(G)****,* ***(H)****,* ***(I)*** *and* ***(J)*** *were all considered as correct curves and quantitative results are detailed in* ***Table 1****.*

***(K)*** *and* ***(L)*** *represent amplification curves of stillborn and adult mice from which quantitative results are detailed in* ***Table 2****.*

**
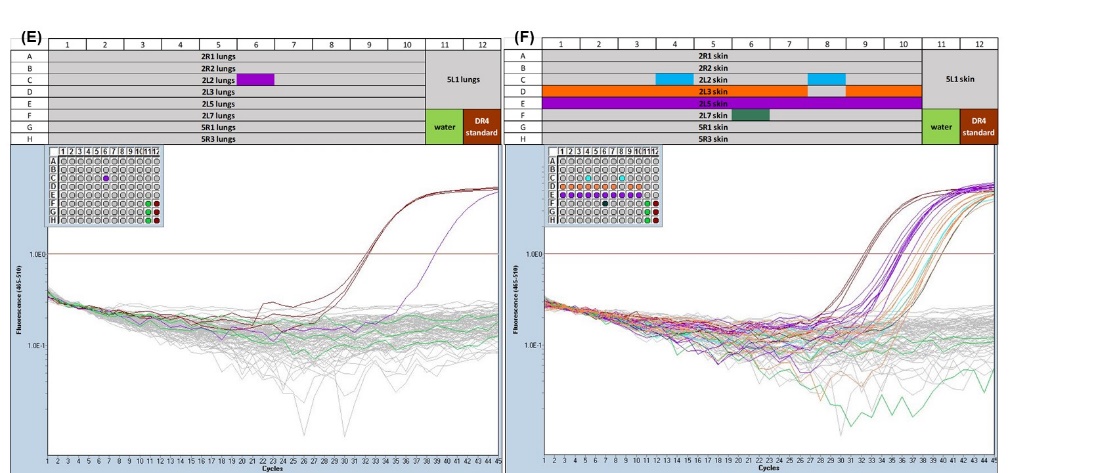
**

**
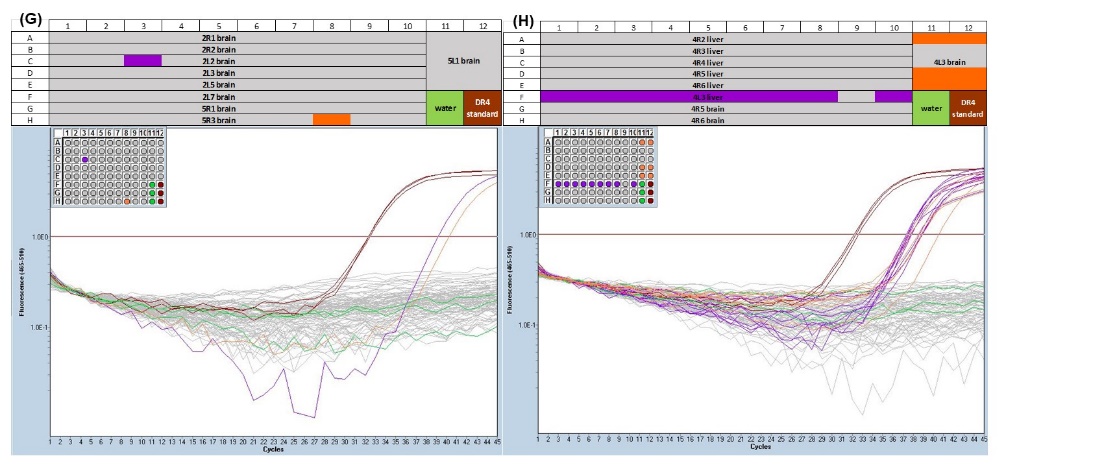
**

**Continued Figure S2. Amplification curves of microchimeric HLA-DR4 positive samples.**

**
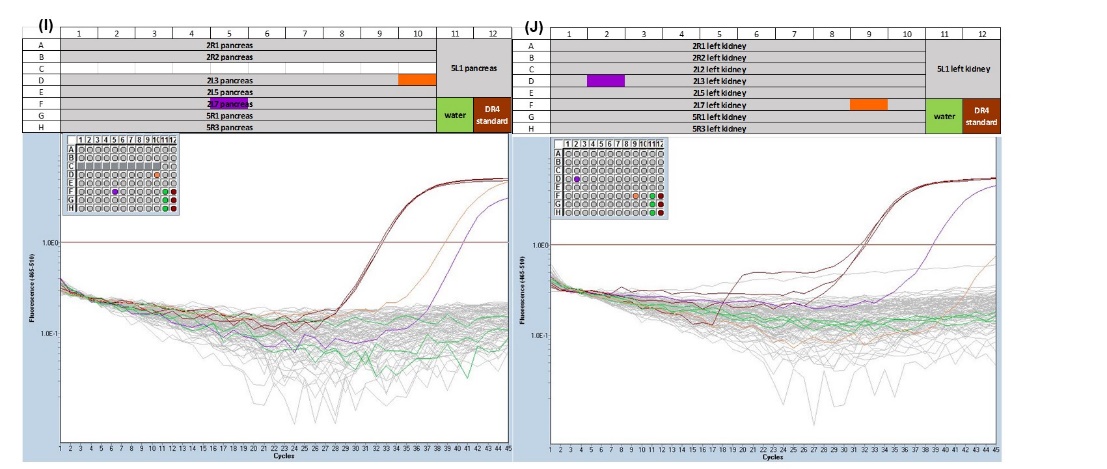
**

**
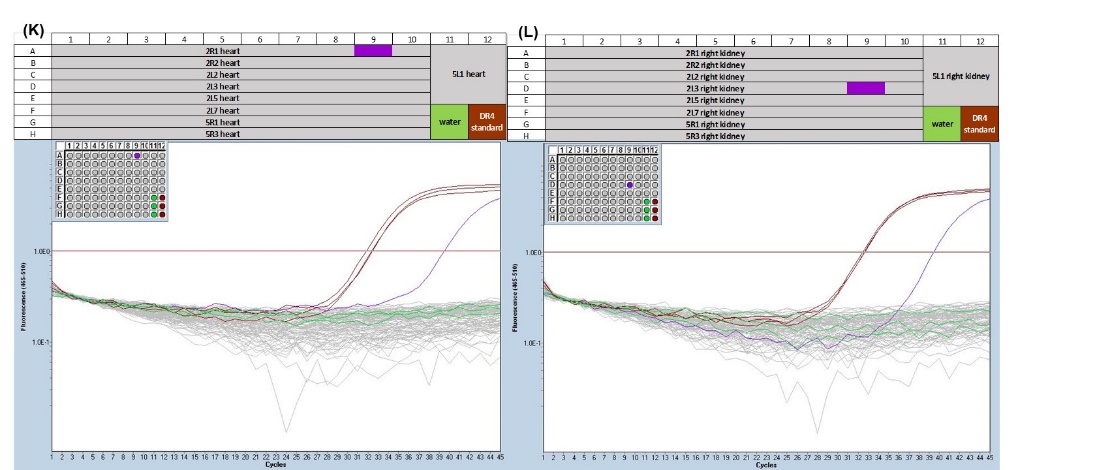
**

**Continued Figure S2 and end. Amplification curves of microchimeric HLA-DR4 positive samples.**

**
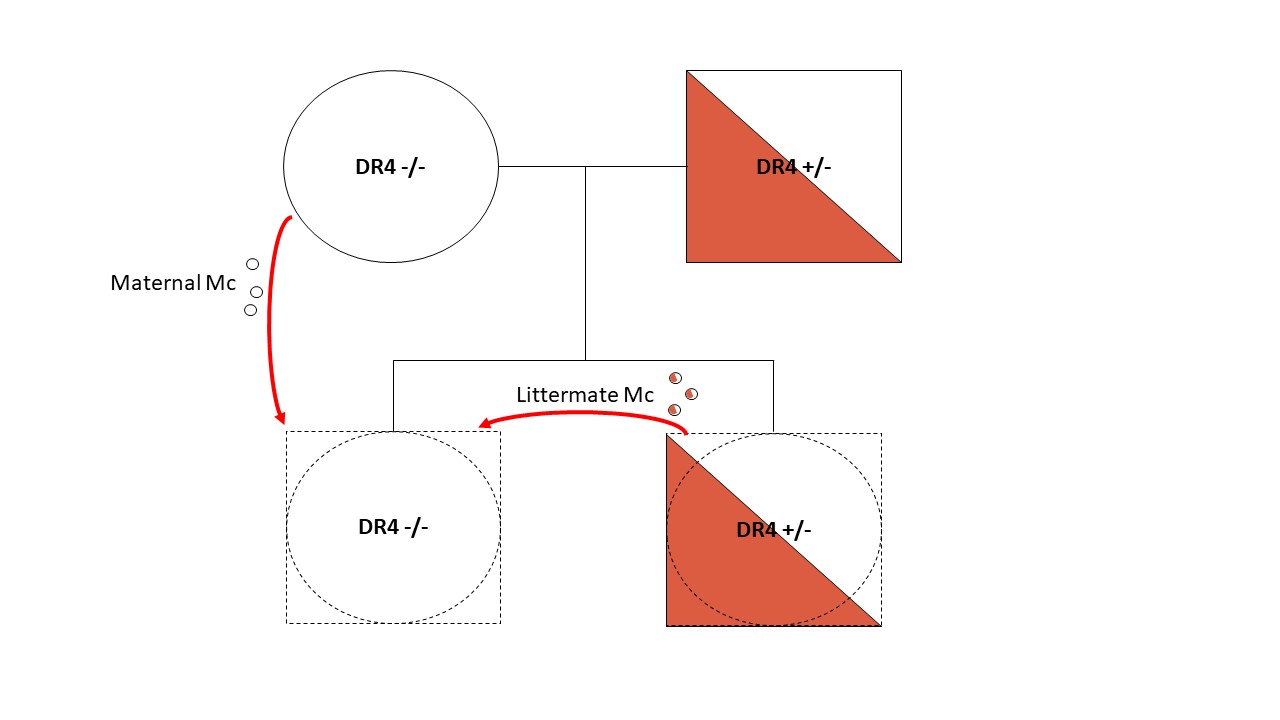
**

**Figure S3. Crosses suitable for obtaining a littermate Mc distinct from the maternal Mc by HLA-DR4-QPCR**

DBA/2 females (H2^d/d^) were crossed with heterozygous B6-DR4D2F1 males (H2^d/b^ DR4^+/-^) thus that in gestational DBA/2 females, the only source of DR4^+^ Mc received by the DBA/2 (H2d/d DR4^-/-^) embryos comes from nearby DR4^+/-^ littermates.


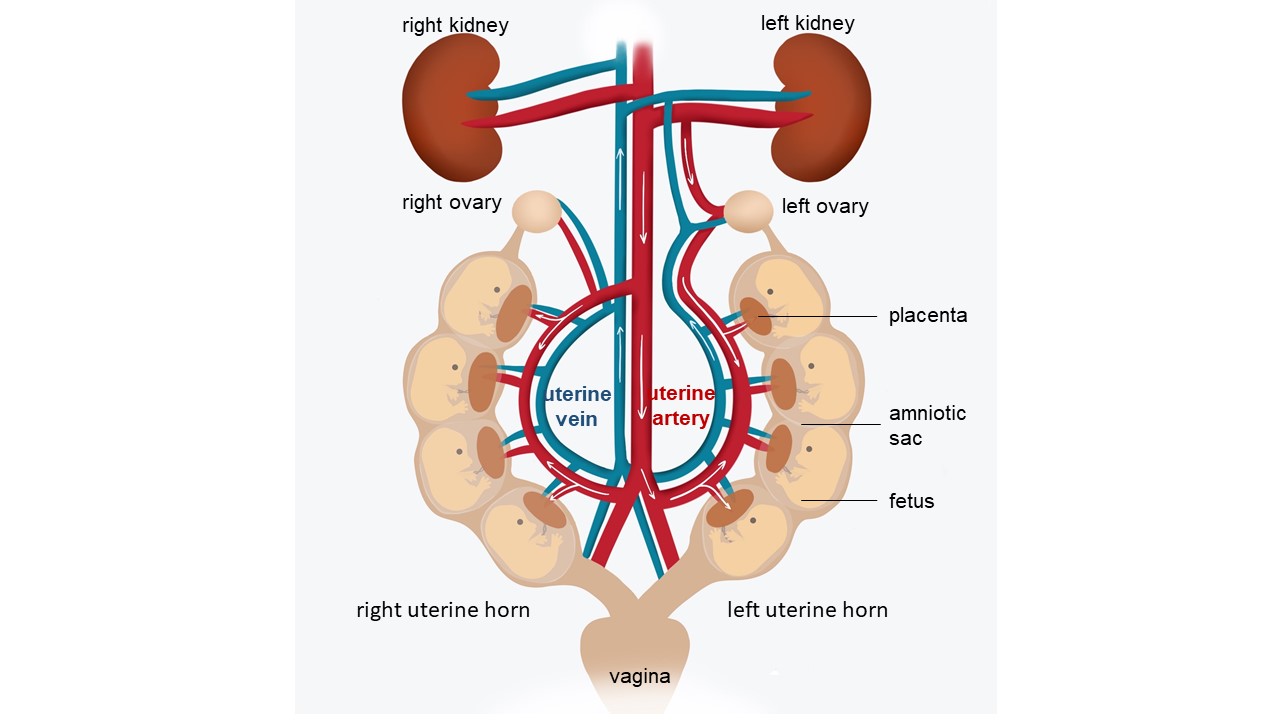


**Figure S4.** **Schematic figure depicting the uterine horns and loop vessels in a pregnant mouse near the end of pregnancy adapted from Vom Saal et al. *(23).***

Vom Saal et al. using intracardial injection of carbon dyes showed that the circulatory flow of the uterine artery and the uterine vein in mice is bidirectional with a rostral flow coming to vascularize the fetuses in the highest position and a caudal flow coming to vascularize those in lowest position. Both flows generally meet in the middle of the loop, depending upon the number of fetuses to vascularize.

| **Group #** | **Tissue** | **Mean quantity of cells tested (in gEq cells)** | **SD** | **Number of mice** |
| --- | --- | --- | --- | --- |
| 1 | Brain | 177461 | 32968 | 6 |
| 2 | Brain | 193972 | 36187 | 9 |
| 1 | Heart | 175104 | 51778 | 3 |
| 2 | Heart | 209126 | 23406 | 8 |
| 1 | Liver | 182984 | 20881 | 6 |
| 2 | Liver | 184878 | 35165 | 9 |
| 1 | Pancreas | 144980 | 37937 | 2 |
| 2 | Pancreas | 154435 | 39430 | 6 |
| 1 | Skin | 205348 | 60926 | 2 |
| 2 | Skin | 193041 | 34170 | 7 |
| 1 | Placenta | 197151 | 24585 | 6 |
| 2 | Placenta | 171188 | 44999 | 8 |
| 1 | Lung | 228690 | 76727 | 2 |
| 2 | Lung | 242189 | 34635 | 7 |
| 1 | Right Kidney | 177451 | 92924 | 2 |
| 2 | Right Kidney | 187667 | 63855 | 7 |
| 1 | Left Kidney | 140236 | 53242 | 2 |
| 2 | Left Kidney | 155190 | 67240 | 7 |
| 1 | Thymus | 224241 | 45116 | 2 |
| 2 | Thymus | 218074 | 36848 | 7 |
| 1 | Remaining tissue | 214899 | 15917 | 4 |
| 2 | Remaining tissue | 244996 | 87299 | 2 |

**Supplementary Table S1. Mean equivalent number of cells tested for littermate microchimerism for each tissue in both groups.**

Group 1: the group of fetuses with no nearby DR4^+/-^ fetuses, Group 2: the group of fetuses with one or two nearby DR4^+/-^ fetuses in the same uterine horn. Results are expressed as the number of genome equivalent (gEq) of host cells.
